# Impaired Baroreflex Function in an Ovine Model of Chronic Heart Failure Induced by Multiple Coronary Microembolizations

**DOI:** 10.3389/fphys.2019.01420

**Published:** 2019-11-22

**Authors:** Yonis Abukar, Nigel Lever, Mridula Pachen, Ian J. LeGrice, Rohit Ramchandra

**Affiliations:** ^1^Department of Physiology, University of Auckland, Auckland, New Zealand; ^2^Department of Cardiology, Auckland District Health Board, Auckland, New Zealand

**Keywords:** heart failure, embolization, neural control, sheep, heart failure model

## Abstract

Testing new therapies in heart failure (HF) requires a chronic stable model of HF in large animals. Microembolization of the coronary arteries has been used to model HF previously; however, neural control has not been previously explored in this model. Thus the aim of this study was to further characterize neural control in this model of HF. HF was induced by infusion of microspheres (45 micron; 1.3 ml) into the proximal left coronary artery or left descending coronary arteries, with three sequential embolizations over 3 weeks. Twelve to 14 weeks after the final embolization, and when ejection fraction had decreased below 45%, animals were instrumented to record blood pressure and heart rate. Baroreflex control of heart rate was investigated in conscious animals. Additionally, pressure-volume loops were constructed under anesthesia. Embolization-induced HF was associated with a decrease in mean arterial pressure (67 ± 2 vs. 85 ± 4 mmHg, *p* < 0.05), an increase in heart rate (108 ± 4 vs. 94 ± 4 bpm, *p* < 0.05), and a significant increase in left ventricular end-diastolic pressure (11.4 ± 2 vs. 6.2 ± 1 mmHg, *p* < 0.01). Under conscious conditions, there was a significant decrease in the gain (−8.2 ± 2 vs. −4.1 ± 1 beats/min/mmHg, *p* < 0.05) as well as the lower plateau of the baroreflex in HF compared to control animals. HF was also associated with significantly increased respiratory rate (107 ± 4 vs. 87 ± 4 breaths/min, *p* < 0.01) and incidence of apneas (520 ± 24 vs. 191 ± 8 apnea periods >4 s, *p* < 0.05), compared to control sheep. The microembolization model of heart failure is associated with an increase in left ventricular end-diastolic pressure, impaired cardiac function, and altered baroreflex control of the heart. These findings suggest this chronic model of HF is appropriate to use for investigating interventions aimed at improving neural control in HF.

## Introduction

Heart failure with reduced ejection fraction (HFrEF) is characterized by progressive dysfunction of left ventricular muscle, myocyte remodeling, and activation of autonomic and hormonal systems (Jackson et al., 2000; Kemp and Conte, 2012). Despite the advances in therapies and prevention, patients with HF have high rates of morbidity and mortality (Falk et al., 2005; Savarese and Lund, 2017). The balance between the sympathetic and parasympathetic systems, in relation to cardiovascular function, is altered in response to ventricular dysfunction (Amorim et al., 1981; Benedict et al., 1996; Floras, 2009). Specifically, it is recognized that there is abnormal hyperactivity of the sympathetic nervous system (Watson et al., 2007; Ramchandra et al., 2009b) and disinhibition of the parasympathetic nervous system that leads to worsening of the condition (Eckberg et al., 1971; Kinugawa and Dibner-Dunlap, 1995; Bibevski and Dunlap, 1999; Dunlap et al., 2003).

Numerous studies have been conducted on small animal models of HF and these have been instrumental in understanding the pathogenesis of HF. However, there are significant differences between small animal models of HF and human HF beyond scale. These include differences in baseline heart rate, oxygen consumption, and excitation-contraction coupling in cardiac tissue (Haghighi et al., 2003; Dixon and Spinale, 2009). Therefore, a clinically relevant large animal model of HF with similar anatomy and physiology of the heart is crucial. In this context, a number of different large animal models of HF have been studied.

The primary methods to induce HFrEF in large animals include high rate pacing, cardiotoxin infusion, and coronary ligation. The pacing-induced HF model has been used in sheep (Timek et al., 2003; Byrne et al., 2004), pigs (Tanaka et al., 1993; McMahon et al., 1996), and dogs (Prabhu and Freeman, 1995; O’Rourke et al., 1999). The technique of rapid supraphysiological pacing of either the atrium or the ventricle for approximately 4 weeks results in a reproducible model of HFrEF. This model shows similar hemodynamic and mechanical phenotypes as dilated cardiomyopathy seen in human patients with HF. However, one disadvantage of this model is the absence of tissue fibrosis if the pacing is not continued for a prolonged period of time. In addition, left ventricle dysfunction tends to reverse when pacing is terminated, especially if the pacing was performed for short durations (Spinale et al., 1991; McMahon et al., 1996).

Intracoronary or intravenous infusion of the cardiotoxin doxorubicin in dogs (Bristow et al., 1980; Toyoda et al., 1998) and sheep (Chekanov, 1999; Borenstein et al., 2006) leads to cardiac myocyte injury, cell loss, and HF. A major limitation of this method is the variable degree of LV dysfunction that can result, with no reliable dosing strategy to provide a stable model. A further drawback is the potential for systemic side effects and the management of these.

Finally, coronary ligation has been used to induce HF in large animals (Hood et al., 1967). Infarcts larger than 25% resulted in a significant increase in left ventricular end-diastolic pressure and a reduction in stroke volume index. However, mortality using this model can be more than 50%, often as a result of fatal arrhythmias, despite the use of anti-arrhythmic agents or due to severe symptomatic heart failure. Additionally, when the infarction is induced by external coronary ligation rather than percutaneously, post-operative adhesions and fibrosis may make further surgical dissection for subsequent experimental work more difficult with higher rates of complications (Oizumi et al., 1990; Yim et al., 1998; Getman et al., 2006). The combination of extent and impact of infarction with animal loss and surgical access limits the usefulness of this model in the research setting.

Recently, the microembolization model has been used in sheep to develop a chronic model of HF over a period of 3 months. The embolizations have been conducted using injection of either microspheres (Schmitto et al., 2009) or gelfoam (Devlin et al., 2000). The advantage of the microembolization model is that the changes are irreversible and the study with the infusion of microspheres has reported a high success rate of induction of HF; however, mortality rates were high in the gelfoam study. Until now, studies that have examined this model have focused on structural changes in the heart (Huang et al., 2004; Monreal et al., 2004) and putative alterations in neural control have not been examined. Thus, the aim of this study was to further characterize changes in heart function after repeated microembolizations in sheep and examine whether baroreflex control of heart rate is altered in the conscious state.

## Materials and Methods

Experiments were conducted on conscious, adult female Romney ewes weighing 50–80 kg, housed in individual crates, and acclimatized to laboratory conditions (18°C, 50% relative humidity, and 12-h light-dark cycle) and human contact before any experiments. All experiments and surgical procedures were approved by the Animal Ethics Committee of the University of Auckland. The sheep were fed 2 kg/day (Country harvest pellets) and had access to water *ad libitum*.

### Embolization Surgical Procedure

Nine sheep (female, weight: 59 ± 3 kg) underwent three weekly sequential embolizations of either the proximal left coronary artery or left descending coronary arteries. Six control sheep (female, weight: 55 ± 5 kg) without microembolizations were also used in the study. Anesthesia was induced with 2% Diprivan (Propofol) (5 mg/kg IV, AstraZeneca, AUS) and maintained with a 2% isoflurane-air-O_2_ mixture. Sheep were given antibiotic injections (6 ml i.m.; Oxytetra, Phenix, NZ) at the start of the surgery. Additionally, to provide analgesia, sheep were given Ketofen 10% (1 ml i.m.; Merial, Boehringer Ingelheim, NZ) at the start of surgery.

The methods employed have been described before (Schmitto et al., 2009). Once anesthetized and intubated, the sheep were placed in a supine cradled position and four electrodes were inserted into the left and right sides of the sternum and in the hind-limbs near the knee joint, subcutaneously, to record ECG. Recordings were obtained from lead II prior to the infusion of the microspheres and for a further 5 min after infusion. The recordings were made on a dual bio amp electrocardiograph switch box with power lab and LabChart (AD Instruments, NZ). A change in the ST segment (elevation or depression) and T wave (inversion) on one or more limb leads was taken as indication of successful embolization. The left or right femoral artery was accessed percutaneously using an 8F (CORDIS®, USA) sheath. Using an 8F AL2 (CORDIS®, USA) guide catheter under fluoroscopic guidance, the left main coronary artery was then cannulated and the catheter was advanced either into the proximal left coronary artery or left descending coronary arteries. All sheep in the HF group underwent three sequential selective microembolizations to arterial supply of the left ventricle with polystyrene latex microspheres (45 μm; 1.4 ml, Polysciences, Warrington, PA, USA). The three embolizations were each performed 1 week apart, to ensure maximum left ventricle coverage. Prior to each embolization event, β-blocker (metoprolol up to 20 mg/kg, IV) and lignocaine (2 mg/kg, IV) were injected intravenously in order to prevent ventricular arrhythmias. Three sheep did not survive to the second embolization. These sheep showed signs of pulmonary edema but despite diuretics did not recover and were euthanized.

### Echocardiography

Echocardiogram recordings were obtained and analyzed before embolizations and 3 months after the first embolization procedure. This was also done for the group of control sheep. The echocardiogram, using a Hewlett Packard Sonos 1,000, was performed while the sheep were conscious. In the long-axis M-wave echocardiography, diastole, systole, fractional shortening, and ejection fraction parameters were obtained and calculated for the left ventricle.

### Instrumentation Sheep Surgery

After 3 months, once sheep were deemed to have sufficient left ventricle dysfunction (ejection fraction <45%), the animals were instrumented to measure mean arterial pressure (MAP), heart rate (HR), and diaphragmatic electromyography (dEMG) as an index of respiration. The instrumentation procedure was also conducted in a group of control sheep. The procedure for electrodes placement for dEMG has been described previously (Sieck and Fournier, 1990). Two strips of seven-stranded Cooner Wires (AS 633-7SSF, Cooner Wire, CA, USA) were implanted into the diaphragm and secured with silicone gel. To get an index of blood pressure and venous infusion, an incision was made in the neck and a single-tip pressure probe (Millar Inc., Texas, USA) was inserted into the carotid artery. A cannula was inserted into the jugular vein to have an entry point for venous infusion. dEMG measurement was recorded from the pair of electrodes inserted into the diaphragm, with the signal amplified (X10, 000), and filtered (band pass 0.3–3.0 kHz). All the parameters were recorded on a desktop computer with a CED micro 1,401 interface and a data acquisition program (Spike 2).

### Hemodynamics Measurements and Analysis

All recordings were done at least 3 days after instrumentation surgery. Blood pressure was obtained from a pressure probe unit (Millar Inc., USA). Heart rate (HR) was calculated from blood pressure channel. In conscious, standing sheep, HR and MAP were obtained from a 2-h recording and averages were obtained for each animal. To determine the adrenergic effects on hemodynamic parameters and heart rate, β-adrenergic receptor blockade (propranolol, LKT chemicals, USA) was infused (30 mg bolus followed by 0.5 mg/kg/h infusion for 90 min).

### Arterial Baroreflex Control of Heart Rate

In two groups of six conscious sheep, after a 5-min baseline recording of mean arterial pressure and heart rate, baroreflex curves were generated by measuring the responses of heart rate to increasing doses of phenylephrine hydrochloride (25, 50, 100, 200, and 400 mg/min) and sodium nitroprusside (25, 50, 100, 200, and 400 mg/min). For analysis, the baseline blood pressures were sorted from the lowest to the highest pressures and put into bins of 3 mmHg change each. The mean systolic blood pressure of each bin was plotted against the mean HR.

### Plasma Brain Natriuretic Peptide, Epinephrine, and Norepinephrine Measurement

Venous blood samples (10 ml) were collected into an EDTA (BD Vacutainer, NJ, USA) tube. Plasma was rapidly separated with a centrifuge at 4°C at 3,000 rpm, within 5 min of blood collection, and snap-frozen at −80°C. The assays for brain natriuretic peptide (BNP) (Pemberton et al., 1997; Lewis et al., 2017), epinephrine, and norepinephrine (Justice et al., 2015) have previously been described. All samples from individual animals were measured in the same assay (BNP or epinephrine or norepinephrine) to avoid inter-assay variability.

### Diaphragm Electromyogram Analysis

To assess diaphragmatic EMG parameters, resting breathing rate and apnea periods were measured in control and HF animals. Breathing rate average was obtained from dEMG activity in a 12-h baseline period in each animal. Apnea was defined as cessation of diaphragmatic activity. To be considered significant, apnea events had to persist for a minimum of 4 s. To quantify apnea incidence, we calculated an apnea index to indicate the number of apnea periods (>4 s) occurring in a 12-h period.

### Pressure-Volume Loops

The acute pressure-volume loop experiments were conducted at the end of the protocol under anesthesia. To determine the left ventricle pressure-volume relationship, a conductance catheter was placed into the left ventricle through the left carotid artery. This method has been described previously in detail by Baan et al. (1984). Briefly, a 5 s (number 5, straight) seven-electrode conductance catheter that has a micromanometer tip was inserted into the left ventricle *via* a guide cannula, along the longitudinal axis. The catheter was connected to a Millar (Millar Inc., Texas, USA) and AD Instruments pressure-volume processing unit and signals were acquired using LabChart, a data acquisition and data analysis software. Volume correction was done through an estimation, using a hypertonic saline solution (20% salt, 10 ml per infusion) infusion. A cardiac output transonic flow probe was also inserted around the aorta. Briefly, a thoracotomy was performed and fourth rib removed to access the heart. A flow probe was implanted on the aorta (28PS, Transonic Systems, USA) and connected to LabChart to measure cardiac output while performing pressure-volume loops.

Pressure-volume loops were analyzed offline using LabChart (AD Instruments). After volume correction, 10 cardiac cycles at baseline were analyzed. Also, cardiac output recordings from the animal were used for alpha calibrations. Stroke volume, ejection fraction, left ventricular end-diastolic and end-systolic pressure and volume, maximum rate of pressure generation (dP/dt max), peak rate of pressure decline (dP/dt min), and maximum conduction velocity (dV/dt max) were measured.

At the end of these experiments, the sheep were euthanized with an overdose of sodium pentobarbitone (0.5 ml/kg, intravenously) (Provet NZ Pty Ltd., New Zealand). Once respiration and cardiac function had ceased, cardiac tissue was collected for histological analysis.

### Measurement of Cardiac Collagen

At the end of the terminal experiment, the hearts were collected and a portion of the left ventricle wall (specifically left ventricle free wall) was fixed in neutral buffered formalin (Shandon Glyo-Fixx, Thermo Scientific). Tissue blocks were then transferred to 30% sucrose solution. Heart blocks were then cut at 40-μm sections using a cryostat. To measure collagen deposition in the left ventricle wall of the sheep hearts, 12 sections (six control and six HF sheep hearts) were stained with Masson’s Trichrome stain (MTS) solution (Biebrich Scarlet-Acid Fuchsin, PTA/PMA and Aniline Blue). Cardiac muscle fibers stain red and collagen stains blue. Sections were subjected to two washes and then three changes of 100% ethanol and finally one wash of xylene solution before cover slipping.

### Statistical Analysis

All data are expressed as mean ± SEM, except where indicated. The effects of the microembolizations on the baseline levels of MAP, HR, ejection fraction, fractional shortening, respiratory rate, apnea incidence, heart and body weight, collagen fibers deposition, pressure-volume relationship parameters, and the arterial baroreceptor relationships of HR and sysBP were determined using unpaired Student’s *t*-tests (two groups – control vs. heart failure). The effects of the individual microembolization procedures on the baseline levels of ECG (before and after each microembolization) and ejection fraction and fractional shortening (before and 12–14 weeks after microembolizations) were determined using paired Student’s *t*-tests (within-animal). A one-way ANOVA test was used for changes in resting heart rate plotted in 3 mmHg change bins. Data were analyzed using the statistical package SigmaStat (Version 2, Access Softek Inc., 1995). Data were considered significant if *p* < 0.05.

## Results

### Acute Changes After Embolization

**Figure 1:**
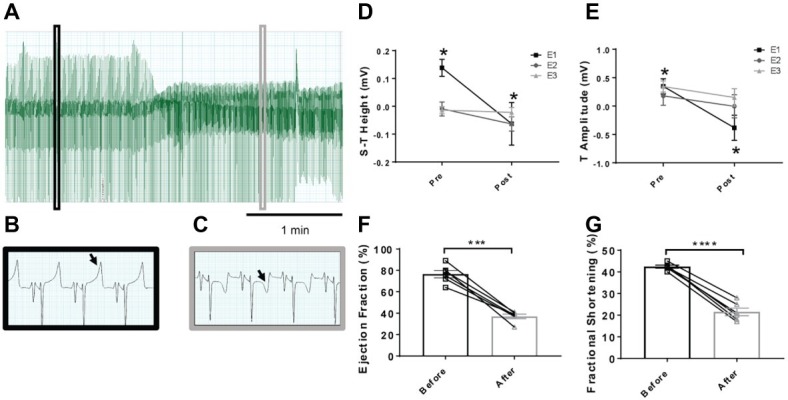
Representative electrocardiogram traces in sheep before and after microembolizations and mean data on S-T and T-wave segments. **(A)** Lead II trace highlighting the changes over time in the ECG, with **(B)** highlighting magnified lead II activity before microembolization and **(C)** after microembolization. Arrows highlight the T-wave of the ECG, showing a depression after microembolization. **(D)** Mean data on the height of the S-T segment and **(E)** T-wave amplitude before and after the individual microembolization procedures. Pre- indicates before microembolization and post- after microembolization. Microembolization 1 (E1) represented by square, microembolization 2 (E2) by circle, and microembolization 3 (E3) by triangle. **(F)** Ejection fraction before (open square) and after (12–14 weeks after) embolizations (open triangle) in the same animals and **(G)** fractional shortening before (open square) and after (12–14 weeks after embolizations) (open triangle). ^*^p < 0.05 in E1 only, *n* = 6, ^***^p < 0.0001 in ejection fraction, ^****^p < 0.0001 in fractional shortening, *n* = 6.

Embolization of the coronary artery at the first time point resulted in a significant change in the lower S-T segment height and also the T-wave amplitude (*p* < 0.05; Figure 1). There were, however, no further decreases in this parameter after embolization procedures 2 and 3. The baseline height of the S-T segment was significantly lower after the second and third embolization procedure compared to the first (*p* < 0.05). These differences in embolization procedures 2 and 3 compared to embolization 1 were also observed in the T-wave amplitude.

### Resting Hemodynamic Variables

**Figure 2:**
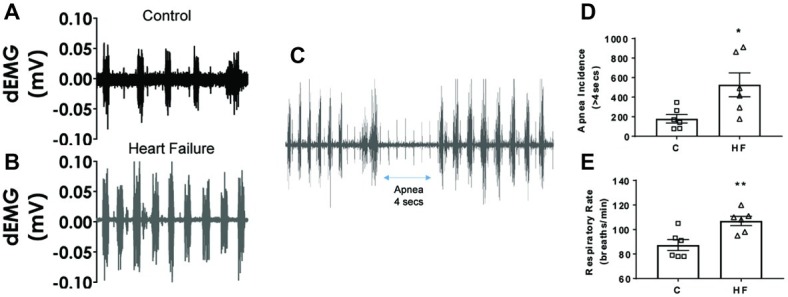
Raw traces of sheep diaphragmatic electromyography and respiration rate in control and heart failure sheep. **(A)** Raw signal of dEMG in a control sheep and **(B)** raw signal of dEMG in a HF sheep, and **(C)** raw signal showing an apnea segment in a HF sheep 16 weeks after embolization. **(D)** Apnea incidence and **(E)** respiratory rate in control healthy sheep and in HF sheep 16 weeks after microembolization, control (square) and HF (triangle). ^*^p < 0.05, ^**^p < 0.01. Bars represent mean values ± SEM. Data from individual sheep are illustrated with symbols; dEMG, diaphragmatic electromyography.

The resting levels of hemodynamic variables in the control and HF sheep are shown in Table 1 and Figure 1. Ejection fraction and fractional shortening data in Figure 1 are within animal and the rest of the data is a comparison between instrumented control and HF sheep. Repeated microsphere infusions into the coronary vasculature resulted in significantly lower ejection fraction (*p* < 0.001) and fractional shortening (*p* < 0.0001) in HF sheep, after 12–14 weeks (Figure 1). HF was associated with a significant decrease in MAP (67 ± 2 vs. 85 ± 4 mmHg, *p* < 0.01), and an increase in heart rate (108 ± 4 vs. 94 ± 4 bpm, *p* < 0.05) compared to the control animals. Plasma levels of norepinephrine and brain natriuretic peptide were also significantly higher (*p* < 0.05) (Table 1). The respiratory rate was significantly higher in the HF sheep (109 ± 4 vs. 88 ± 5 breaths/min, *p* < 0.01) (Figure 2). The HF sheep showed more incidences of temporary cessation of breathing in a 12-h cycle (525 ± 123 vs. 179 ± 44 apnea periods longer than 4 s, *p* < 0.05, Figure 2). Infusion of propranolol reduced heart rate more in the HF group compared with control (9 ± 1 vs. 3 ± 1, bpm, change in HR before and after propranolol, *p* < 0.001) and there was no significant change in MAP between the groups after propranolol infusion.

**Table 1:** Resting values for hemodynamic parameters between conscious normal and heart failure sheep.

Parameter	Normal (*n* = 6)	Heart failure (*n* = 6)
Ejection fraction	79 ± 1	39 ± 2^†^
Fractional shortening	43 ± 1	20 ± 1^†^
Heart rate, beats/min	94 ± 4	108 ± 4^*^
Mean arterial pressure, mmHg	85 ± 4	67 ± 2^**^
Body weight (kg)	56 ± 2	70 ± 3^**^
Brain natriuretic peptide (pmol/L)	2.20 ± 0.12	3.79 ± 0.52^*^
Epinephrine (pmol/L)	380 ± 73	751 ± 188
Norepinephrine (pmol/L)	3,272 ± 901	10,628 ± 2509^*^

* p < 0.05;

** p < 0.01;

† *p < 0.0001*.

**Figure 3:**
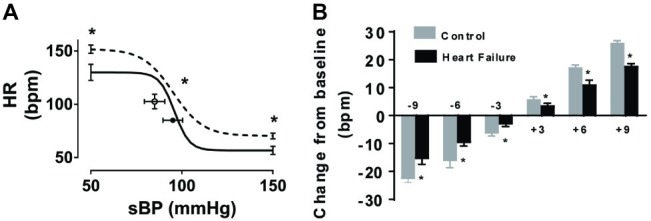
Effect of microembolizations on the arterial baroreflex in conscious healthy and HF sheep. **(A)** Control animals (solid line) and HF animals (dashed line) (*n* = 6/group). Error bars at each end of the curves refer to ±SEM of the upper and lower heart rate (HR) plateaus. Circles and error bars represent mean ± SEM of resting systolic blood pressure (sBP) and heart rate (HR). **(B)** Changes in resting heart rate, due to phenylephrine and sodium nitroprusside infusions, plotted in 3 mmHg change bins. Gray bars represent control animals, black bars represent heart failure animals. ^*^p < 0.05, *n* = 6/group.

To determine whether repeated micro-embolism induced any impairment in baroreflex control, we assessed arterial baroreflex function in both control and HF sheep. The baroreflex relationship showed a significant difference in the maximum gain (−8.2 ± 2 vs. −4.1 ± 1 beats/min/mmHg, *p* < 0.05), as well as the upper (152 ± 4 vs. 130 ± 8 beats min^−1^, *p* < 0.05) and lower (70 ± 3 vs. 57 ± 4 beats min^−1^, *p* < 0.05) plateaus of the HR baroreflex curve (Figure 3A). To determine the responses in HR over the operating range of the curve, we examined the differences in the mean resting HR values close to the resting MAP levels. HR changes from baseline, in response to phenylephrine and sodium nitroprusside infusions, plotted in 3-bpm bins highlight the significantly decreased response in the HF animals (Figure 3B) suggesting an impaired gain of the baroreflex curve at the operating ranges in these animals.

### Pressure-Volume Loops Under Anesthesia

The ejection fractions calculated from the pressure-volume loops under anesthesia showed significantly lower ejection fraction compared to the conscious condition in both groups of sheep (*p* < 0.01); however, the difference between the groups remained intact. Interestingly, there was no change in baseline cardiac output between the normal and the HF sheep. The LV end-diastolic pressures and volumes in the HF sheep were significantly higher, similar to end-systolic volume (Table 2). The maximum rate of pressure generation tended to be higher in the control animals; however, this was not significant.

**Table 2:** Pressure-volume loops testing left ventricular function in anesthetized normal and heart failure sheep.

Parameter	Normal (*n* = 6)	Heart failure (*n* = 6)
Ejection fraction (%)	56 ± 7	25 ± 4^**^
Cardiac output (L/min)	2.8 ± 1	3.9 ± 1
Stroke volume (ml)	33 ± 6	42 ± 6
End-diastolic volume (ml/min)	74 ± 21	194 ± 36^*^
End-systolic volume (ml/min)	40 ± 20	154 ± 34^*^
End-diastolic pressure (mmHg)	6.2 ± 1	11.4 ± 2^**^
dP/dT max (mmHg/s)	1,745 ± 321	1,257 ± 154
dV/dT max (ml/s)	1,425 ± 155	3,361 ± 617^**^

* p < 0.05;

** *p < 0.01*.

### Collagen Deposition

**Figure 4:**
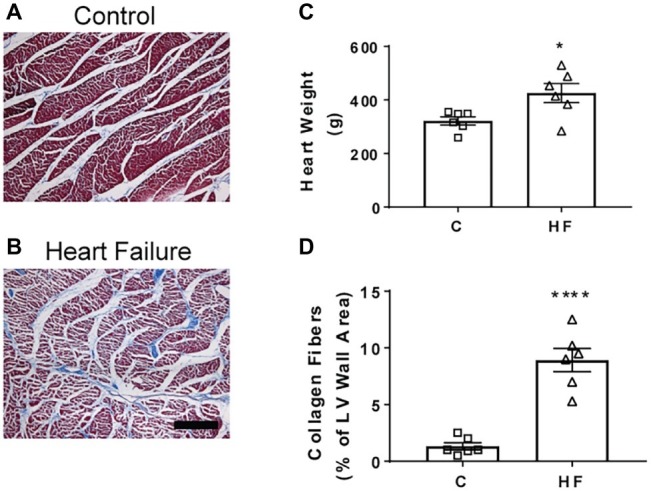
Representative sheep cardiac tissue images and effect of microembolizations on collagen deposition and heart weight. **(A)** Top image shows section of left ventricle wall of the sheep heart in a control healthy animal and **(B)** bottom image shows section of left ventricle wall in a HF sheep 16 weeks after microembolization. **(C)** Heart weights of control (square) and HF (triangle) sheep. **(D)** Collagen content in control and HF sheep, ^*^p < 0.05, ^****^p < 0.0001. Bars represent mean values ± SEM. Data from individual sheep are illustrated with symbols. C, control (square); HF (triangle), heart failure; LV, left ventricle of heart. Scale bar = 100 μm.

Microscopically, there was a significant degree of ischemic damage seen in the hearts of the HF sheep. Left ventricular collagen deposition in the HF sheep was determined as an indicator of cardiac damage. The mean left ventricular collagen content in the HF sheep was significantly higher than in healthy control sheep (8.9 ± 1 vs. 1.3 ± 0.3% of LV wall area, *p* < 0.001). The wet heart weight of the sheep in HF was also significantly greater than that of the control sheep (426 ± 35 vs. 322 ± 15 g, *p* < 0.05, Figure 4).

## Discussion

We have established a reliable model of heart failure with reduced ejection fraction by repeated microembolizations of left ventricular coronary arteries. Our study has a number of new findings not reported previously: (1) there is a depression in the gain of the baroreflex in this model of HF; (2) diaphragmatic electromyography showed increased respiratory rate in the HF animals compared to the control animals, suggesting increased inspiratory effort; (3) in addition to an increase in respiratory rate, there was an increased incidence of apneas in this animal model. Taken together, our findings suggest that this model of HFrEF replicates the neural and respiratory instabilities seen in human HF.

One of the main clinical signs of a myocardial infarct is changes in the ECG, namely changes in the S-T segment and the T-wave. For the S-T segment, in most cases, patients that have had a suspected myocardial infarct present with an elevation in this segment (Members et al., 2012). In contrast to clinical findings, we observed a consistent depression in the S-T segment of the embolized animals. Clinically, patients exhibiting an S-T depression had adverse long-term outcomes (Schechtman et al., 1989; Krone et al., 1993; Hyde et al., 1999). This depression in our study may reflect the recordings being done in a supine cradled position for these sheep. In the absence of pre-cordial leads, it makes it difficult to draw conclusions as to which region of the left ventricle received most ischemic damage.

Several other studies, in patients, have shown collagen deposition in the myocardium, suggestive of ventricular remodeling (Weber et al., 1993; Martos et al., 2007; Mewton et al., 2011). The gross and microscopic changes of myocyte hypertrophy and increased collagen fibrosis have also been demonstrated in other models of HF (Falk et al., 2005). After an episode of myocardial infarction, there is structural remodeling that is initiated by an inflammatory response (Sun, 2008; Suthahar et al., 2017). Scarring then forms at the infarcted area. While the scarring and fibrosis at the site of infarct maintain the structure and integrity of the heart, this remodeling can have consequences for ventricular contractility (Brilla and Weber, 1992).

One of the ways to assess the degree of HF is the construction of pressure-volume loops. One of the advantages of constructing pressure-volume loops is the measurement of parameters independent of pre- and after load (Burkhoff, 2013). A hallmark of systolic dysfunction with reduced ejection is an increase in left ventricular end-diastolic pressure (LVEDP), which was observed in the present study (Table 2). Since LVEDP is a reflection of ventricular performance, the increases in LVEDP may be associated with the size of the infarct or the amount of damage to the left ventricle (Mielniczuk et al., 2007). Furthermore, we also see a significantly higher end-diastolic volume in the HF animals similar to findings in other studies (Sabbah et al., 2000; Morita et al., 2002; Falk et al., 2005). Additionally, there was a significant increase in both end-diastolic (EDV) and end-systolic volume (ESV) in the HF animals. In HF, the severity of an increase in both these volumes at time of referral is a prognostic indicator of mortality in patients (Diaz et al., 1987).

In the present study, we show significant increases in plasma levels of brain natriuretic peptide (BNP) and norepinephrine (Table 1). These increases in plasma levels of BNP (Sakurai et al., 2003) and norepinephrine (Cohn et al., 1984) have been shown previously to relate to the severity of HF. Our findings are in agreement with other models of HF, which have also found elevated plasma levels of norepinephrine and brain natriuretic peptide (Sabbah et al., 2000; Morita et al., 2002).

We report a significant decrease in the MAP of the animals with HF. One mechanism that serves to maintain BP is the arterial baroreflex and previous studies have reported a dampening of the sensitivity of the arterial baroreflex control of heart rate in HF (Eckberg et al., 1971; Ferguson et al., 1992; Grassi et al., 1995) as well as in clinical patients (Mortara et al., 1997). We hypothesized that this model would be associated with a blunted baroreflex and observed an impaired HR response to changes in MAP. This impaired baroreflex is similar to previous reports by us in a pacing-induced model of HF (Watson et al., 2007; Abukar et al., 2016) as well as other models (Higgins et al., 1972; Greenberg et al., 1973; Zucker et al., 1977). In addition to depressed baroreflex function, we also examined sympathetic control of heart rate in this model. We observed a significant decrease in heart rate in the HF animals after beta blockade. This suggests there is a significant contribution in HF animals from the sympathetic nervous system, more so than the control animals in which no significant decrease in heart rate was observed after propranolol infusion. These findings are consistent with our previous studies suggesting low levels of cardiac sympathetic drive and cardiac norepinephrine spillover in normal animals. Both these variables are elevated in animals with pacing-induced HF (Ramchandra et al., 2009a, 2018).

In addition to changes in hemodynamic parameters, we also observed an increase in the breathing rate and an increase in the incidence of apneas. Chronic hyperventilation has been reported in HF patients (Sullivan et al., 1988) and pulmonary congestion is suspected to be the main cause of this hyperventilation (Sullivan et al., 1988). The animals in the HF group had significantly increased body weight compared to the control animals (Figure 1), which points toward fluid congestion. We also saw signs of fluid congestion in the heart and ascites in the HF group at the time of post-mortem although this was not systematically investigated. We speculate that this fluid congestion contributed to the hyperventilation in these animals. In addition to peripheral causes, central apneas are also common in heart failure, with around 40–50% of patients being affected (Javaheri et al., 1995; Vazir et al., 2007). Central sleep apnea can be a marker of HF severity, with central apnea patients showing more advanced symptoms (Oldenburg et al., 2007). Together, these results suggest that the microembolization model replicates the respiratory imbalance seen in HF.

We report successful induction of heart failure in sheep using repeated percutaneous injection of microspheres into the coronary arteries. This approach targets the coronary circulation and thereby circumvents potential systemic effects if a toxin is administered intravenously. Since the HF is induced by a durable ischemic insult, interventions with novel pacing paradigms can be undertaken in pacing naïve hearts, avoiding the potential confounder of pacing-induced changes. Practically, repeated access to the arterial circulation is an important consideration. Previous studies have constructed carotid artery loops to allow repeated access. We percutaneously accessed the femoral arteries and following embolization, digitally compressed the puncture site for hemostasis without the use of a closure device, returning the animal to the crate/pen without any indwelling cannulae retained for future access. This has a number of advantages over other methods of surgical approach to perform the microembolization. Cardiac catheterization using these techniques requires access to fluoroscopy to guide cannulation and confirm successful and stable engagement in the coronary vessels.

There are a few limitations to this approach as well. One important limitation of this approach is the diffuse pattern of blockage and fibrosis that is observed. As such, it is difficult to quantify the region of infarcted tissue adequately as would be possible with coronary ligation. We utilized ejection fraction and fractional shortening from the echocardiographic data to quantify the decline in heart function. In addition, three out of the nine sheep did not survive the first embolization as they developed pulmonary congestion that could be resolved using diuretics. In addition to these three animals, two of the animals studied also needed administration of diuretics after the embolization procedure, and in these two cases, the congestion was resolved and the animals continued in the protocol. While the presence of congestion makes this model clinically relevant, this does necessitate increased animal numbers to complete a cohort of animals for a study.

In conclusion, our study indicates that chronic heart failure can be successfully induced in sheep using repeated injection of microspheres into the coronary arteries. After 2 months, the animals develop clinical signs of heart failure. There is marked increase in heart weight with histological evidence of ventricular fibrosis. There is a decrease in mean arterial pressure and an increase in incidence of apneas. Furthermore, pressure-volume loops show altered left ventricle dynamics in the heart failure sheep and the baroreflex challenge under conscious conditions showed a significant decrease in the gain. We conclude that this is a good model of HF to test changes in either neural control or respiratory function after interventions.

## Data Availability Statement

The data gathered in this study are available upon request to the corresponding author.

## Ethics Statement

The animal study was reviewed and approved by The University of Auckland Animal Ethics Committee.

## Author Contributions

YA, NL, MP, IL, and RR collected the ECG data for this study. All other data were collected by RR, MP, and YA. RR supervised the study. YA completed the data analysis and wrote initial manuscript. All authors contributed in revising the manuscript toward the final version.

### Conflict of Interest

The authors declare that the research was conducted in the absence of any commercial or financial relationships that could be construed as a potential conflict of interest.
